# Modified Rotationplasty as a Composite Free Flap for Femur and Thigh Reconstruction

**DOI:** 10.29252/wjps.10.2.115

**Published:** 2021-05

**Authors:** Ammaar MA Abbasi, Rayaad C Hosein, Rhett N Willis Jr, Valdis M Lelkes, Kevin B Jones, Jayant P Agarwal

**Affiliations:** 1 Medical College, Aga Khan University, Karachi, 74800, Pakistan; 2 Division of Plastic & Reconstructive Surgery, University of Texas Health San Antonio, MC 7844 San Antonio, Texas, United States; 3 Charlottesville Plastic Surgery, 1410 Incarnation Dr. Suite205A, Charlottesville 22901, Virginia, United States; 4 Department of Orthopedics, Oncology Surgery, University of Utah School of Medicine, Salt Lake City 84132, Utah, United States; 5 Department of Surgery, Division of Plastic Surgery, University of Utah School of Medicine, Salt Lake City 84132, Utah, United States

**Keywords:** Rotationplasty, Cortical reorientation, Gait mechanics

## Abstract

Knee rotationplasty is a suitable reconstructive and limb salvage procedure for infected femur and knee prostheses. It involves external rotation of the lower limb with an intact neurovascular bundle to function as a knee joint. Functionally, it has better outcomes when compared to alternate options like above knee amputation. It results in better cortical reorganization and superior stance mechanics, enabling a more efficient gait and better quality of life. Here we report a 57-yr-old male who underwent modified rotationplasty for an infected knee endoprosthesis as a composite lower leg free flap.

## INTRODUCTION

Rotationplasty is a limb sparing surgical technique allowing the ankle to function as a knee joint^1^. It is essentially an intercalary amputation of the knee joint with a 180° axial rotation of the distal limb and attachment to the proximal femur^1^^, ^^2^. 

The procedure was introduced to treat a patient with tuberculosis with a shortened limb. This was later modified to treat proximal femoral focal deficiency in children^3^. In the 1970s it was utilized for reconstruction for distal femoral tumor defects and as an alternative option for above knee amputations^1^^, ^^4^. Rotationplasty has allowed surgeons to perform en bloc resection of the distal femur and proximal tibial tumors with wide margins while preserving the limb, especially in children^3^^, ^^5^. It provides patients with a biological, functional joint which has normal motor control, sensation, proprioception and is better suited for future custom made below knee prostheses^1^^, ^^5^. However, limited data is available regarding its indications, technique and outcomes, especially in adult patients^2^^, ^^6^. 

The principles of rotationplasty are based on preserving an intact neurovascular bundle that supplies the skin, fascia and muscles of the lower leg. While some studies have suggested a modified approach of transection and re-anastomosis of the vessels, this is rarely reported in the literature ^2^^, ^^7^. The reasons reported for this include a prolonged operative time and high risk of anastomotic failure, leading to higher morbidity^1^^, ^^2^. 

Here we report a patient who underwent modified rotationplasty for an infected knee endoprosthesis as a left lower leg composite free flap for thigh and femur reconstruction.

## CASE PRESENTATION

**Fig. 1:**
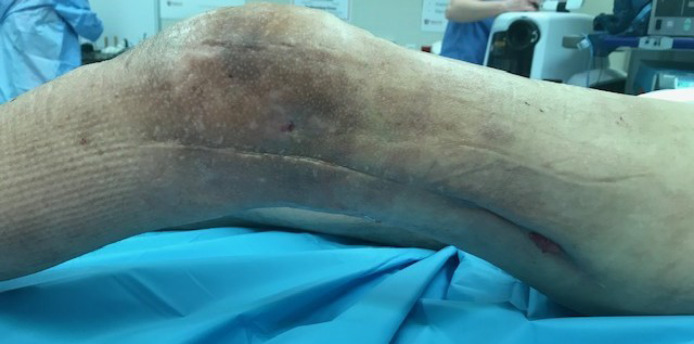
Preoperative view of the multiple draining sinuses, prior effects of radiation and extensive surgical scar

A 57-year-old male presented to our clinic at Huntsman Cancer Institute, Salt Lake City, Utah, US, with history of left popliteal myxoid liposarcoma for which he had undergone external beam radiotherapy, surgical resection and brachytherapy. Despite multiple surgeries for distal femur fractures, he ultimately developed non-union and chronic pain. Distal femur removal and endoprosthetic knee replacement was performed. Later, he developed severe heterotopic ossification, poor lymphatic drainage and eventually multiple infected draining sinuses (Figure 1). A plan was made to completely remove the hardware and amputate at the level of the discharging sinuses. He was referred to us for reconstruction of the residual limb length with a composite flap using the below-knee tissues including the skin, muscle, tibia and fibula. 

Informed surgical and photographic consent was obtained and all information was deidentified. After general anesthesia, the patient was placed in a supine position and a circumferential incision was made proximally over the anticipated femur osteotomy site. The anterior compartment was dissected to reach the shaft of the femur. The infected hardware was removed. Distally, an anterior incision over the knee joint was made to remove the knee and tibial endoprosthesis. 

**Fig. 2:**
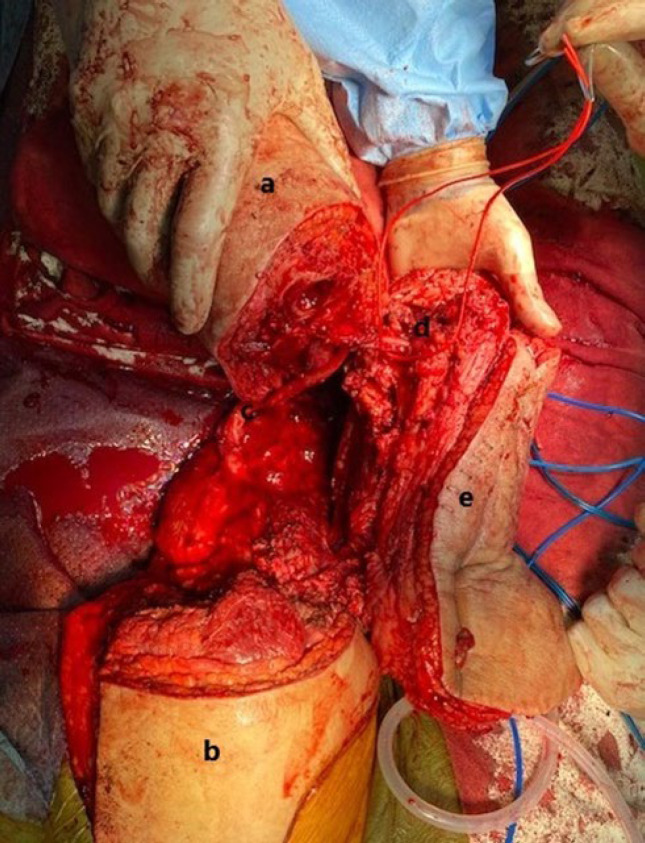
View of structures prior to resection of vessels and intervening segment

The neurovascular bundle of the leg was identified and dissected in a fashion consistent with conventional rotationplasty. The remaining posterior thigh musculature was transected and a large thigh skin flap along with the sciatic nerve was maintained. Prior surgeries and chronic infection had resulted in excessive scar tissue formation, encasing the femoral and popliteal vessels, as shown in Figure 2. Preserving these vessels was an unfeasible option.

**Fig. 3:**
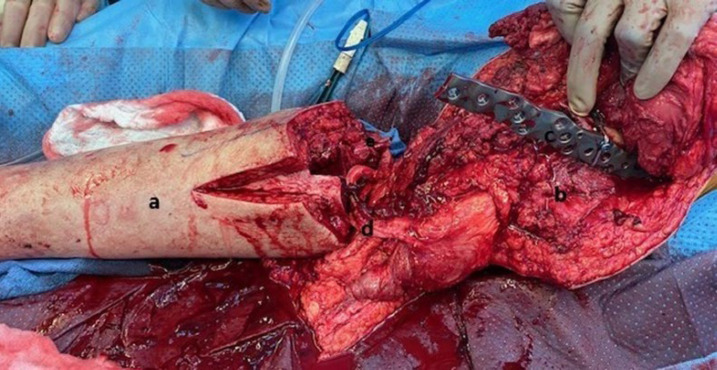
View of structures prior to anastomoses of vessels and osteosynthesis

Once the femur was prepared, the profunda femoris vessels and greater saphenous vein were ligated and superficial femoral and popliteal vessels were transected. The intervening segment was removed (Figure 3). The lower limb was rotated 180º externally, ensuring a satisfactory limb position. The tibia was then secured to the lateral femoral shaft and the vascular anastomosis was performed between the popliteal artery and superficial femoral artery and the greater saphenous vein ends. A Chopart foot amputation was performed and the plantar foot surface was maintained as a healthy surface for future weight bearing. Proximally, the posterior thigh skin flap was rotated to close the lateral defect and the incisions were closed in layers. 

The early post-operative course was uneventful. On postoperative day 5 he was able to walk using crutches and was discharged the next day. At one month, he had been able to maintain baseline movement at the hip joint, a well perfused extremity and intact stump surface sensation.

## DISCUSSION

Infection associated with tumor prostheses is a serious and common complication. Rotationplasty as a limb salvage procedure for such conditions has proven to be a successful option^1^^, ^^8^. It allows the patients to maintain limb length leading to a more active and efficient gait, with less energy consumption during ambulation, less post-operative pain and a better quality of life^1^^, ^^4^^, ^^8^. 

Conventionally, the neurovascular bundle is carefully coiled and placed between tissues on the medial aspect of the femur^1^^, ^^6^^, ^^7^. However, there is a risk of impaired blood flow to the distal limb, secondary to kinking and thrombosis of the vessels especially in patients with chronic infection or history of prior surgery or radiation^2^. 

 In our patient, extensive scar tissue had encased the popliteal and femoral vessels within the intercalary segment. Thus, a conventional rotationplasty was not a suitable option. The reported rates of vascular compromise following rotationplasty with vascular reconstruction range between 11.5 to 15%^2^. However, despite the increased risk of thrombotic complications, the only option for enhancing limb length was to perform a vascular reconstruction in conjunction with a spare parts component. This allowed en bloc removal of the intercalary segment and resection of excess vessel length, thereby, reducing the tortuosity of the pedicle and risk of thrombosis^7^. 

The alternate option would have been a very high above knee amputation with prostheses. However, cortical reorientation and gait parameters in such patients are poor when compared to rotationplasty^9^^, ^^10^. Rotationplasty patients have similar range of motion at the hip, pelvis and knee joints compared to that in normal subjects^9^^-^^11^. However, the range of motion and torque generated at the ankle joint of the prosthetic foot is significantly reduced leading to overuse and strengthening of the unaffected limb^10^^, ^^11^. The overall electromyographic activity of the lower leg muscles showed good coordination with the uninvolved limb during the stance and swing phase. This indicates functional adaptation of the lower leg muscles for their new task^11^. 

The significantly higher knee excursion of the rotated limb in rotationplasty patients accounts for difference in gait outcomes^9^. In above knee amputees, the prosthetic knee is locked in extension and normal early stance phase flexion is absent^12^. This is primarily to maintain the ground reaction force in front of the knee in order to prevent collapse. Since rotationplasty patients have active control of their knee, they are not concerned about their knee position^9^. Furthermore, in above knee amputees weakened hip abduction, excessive adduction and inadequate torque generation at the hip joint on the affected side leads to an unstable gait^9^^, ^^13^. Rotationplasty patients have intact foot proprioception^9^. This coupled with preserved flexion-extension of the affected knee joint and active control results in better functional outcomes and ambulation^10^^, ^^12^.

Outcome differences can also be attributed to the cortical and subcortical reorganization that occurs in above knee amputees and rotationplasty patients. Absence of afferent information following amputation results in reorganization of contralateral sensorimotor regions of the brain. Lower limb amputees have decreased functional connectivity between the contralateral sensorimotor cortex and sub cortical motor regions involved in coordinated controlled movement of the lower limb^14^. Previous studies have also mentioned modifications in the cerebellar gray area, reduction in thalamic volume and a disrupted basal ganglia-thalamus-cortex pathway in these patients^14^. 

A study ^15^ in rotationplasty patients found that motor evoke potentials were higher for the operated limb when compared to healthy controls, but smaller in comparison to the unaffected limb of the patients. This indicated cortical neuroplasticity with increased excitability in the ipsilateral cortex. The results reflected strengthening of the unaffected leg, increased cross education and skill training of the rotated leg. This showed functional adaptation of the rotated limb, which was also seen in electromyographic studies^11^^, ^^15^.

**Fig. 4:**
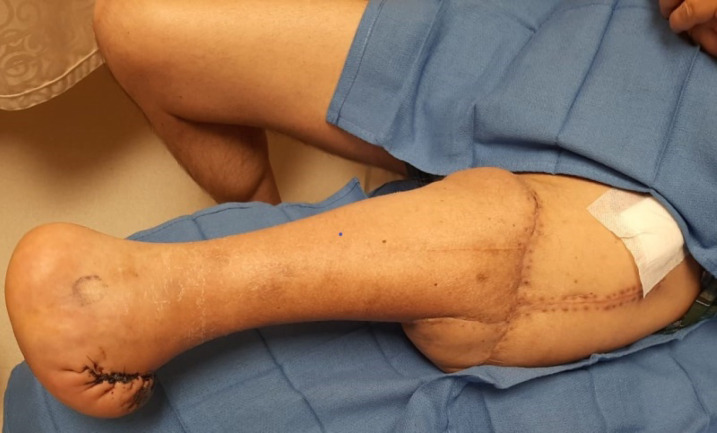
1 month follow up showing a healthy sensate stump at the level of the contralateral knee

Our modified version of rotationplasty provided an adequate sensate stump at the level of the contralateral knee (Figure 4). This will enable the patient to have better tolerance and fitting for future prosthesis^5^^,^^ 8^. Consequently, he is expected to be able to weight bear on the limb with a satisfactory gait. 

a) lower leg b) proximal thigh c) sciatic nerve d) popliteal vessel e) intercalary segment

- lower leg b)posterior thigh flap c) zimmer periprosthetic plate d)sciatic nerve e) transected vessels

## CONCLUSION

Our post-operative outcomes were similar to a conventional rotationplasty patient. Therefore, we could extrapolate that using the lower leg as a spare parts free flap for thigh and femur reconstruction can prove to be a useful technique when combined with the principles of rotationplasty, leading to better limb length and performance with ambulation in the future for adult patients. 
